# Largest case series of giant gallstones ever reported, and review of the literature

**DOI:** 10.1016/j.ijscr.2020.06.001

**Published:** 2020-06-11

**Authors:** Mohammad Al Zoubi, Walid El Ansari, Ahmed A. Al Moudaris, Abdelrahman Abdelaal

**Affiliations:** a Department of General Surgery, Hamad Medical Corporation, Doha, Qatar; b Department of Surgery, Hamad General Hospital, Hamad Medical Corporation, Doha, Qatar; c College of Medicine, Qatar University, Doha, Qatar; d School of Health and Education, University of Skövde, Skövde, Sweden

**Keywords:** Giant gallstone, Large gallstone, Laparoscopic cholecystectomy, Open cholecystectomy

## Abstract

**Introduction:**

Giant/large gallstones have high risk of complications, and technical difficulties during surgery. This case series is the largest ever reported.

**Presentation of cases:**

**Case 1**: Female (44 years), with one year intermittent right upper quadrant colicky pain. Ultrasound: large gallstone (normal gallbladder). Elective laparoscopic cholecystectomy (LC): 6 × 4 × 3.3 cm gallstone. **Case 2**: Female (41 years), presented to emergency room with 3 days right upper quadrant pain/tenderness, vomiting, and positive murphy’s sign. Ultrasound: large gallstone, calculus cholecystitis. Emergency LC: 4.5 × 3.1 × 3.5 cm gallstone. **Case 3**: Male (38 years), with history of gallstones and acute cholecystitis presented with intermittent right upper quadrant pain (2 months) and vomiting. Normal abdominal examination. Ultrasound: large gallstone. Elective LC: 4.1 × 4 × 3.6 cm gallstone.

**Conclusions:**

Gallstones >5 cm are very rare, with higher risk of complications. Gallbladder should be removed even if asymptomatic. Gallstones >3 cm have increased risk for gallbladder cancer, biliary enteric fistula and ileus. LC has challenges that include grasping the gallbladder wall, exposure of Calot’s triangle, and retrieval of gallbladder out of the abdomen. LC appears to be procedure of choice and should be performed by an experienced surgeon, considering the possibility of conversion to open cholecystectomy in case of inability to expose the anatomy or intraoperative difficulties.

## Highlights

• Giant gallstones are very rare and only few cases reported in literature.
• Multiple indications warrants surgery for giant gallstones even in asymptomatic patients.
• Laparoscopic cholecystectomy is the best surgical approach.
• Surgery for giant gallstones carries more technical difficulties and needs expert surgeon.
• Proper pre-operative assessment and planning can decrease peri-operative complications.

## 1 Introduction

Gallbladder stones are very common [1,2]. The size of a gallstone is important, as giant/large gallstones have higher risk of complications and higher technical difficulties during laparoscopic cholecystectomy (LC) [3,4]. Gallstones > 3 cm carry higher risk for gallbladder cancer and gallstones > 5 cm are very rare with only very few cases reported in the literature [[4], [5], [6], [7], [8], [9]]. Gallstones can present in different locations: in the gallbladder which may cause biliary colic or acute cholecystitis, in the biliary tree which may cause biliary obstruction, or in the gastrointestinal tract which may cause gallstone ileus or gastric outlet obstruction in case of large stones. Some authors support that giant gallstones require open cholecystectomy [4], whilst others advocate the laparoscopic approach [5,6].

In the current retrospective single-centre case series at Hamad General Hospital (largest tertiary care hospital) in Doha, Qatar, we review three consecutive cases of laparoscopic cholecystectomy (2017–2020) for giant gallstones measuring 6 cm, 4.5 cm and 4.1 cm in diameter respectively, all located in the gallbladder and we report them in line with the PROCESS criteria [10]. Patients were included if there was a giant gallstone (> 4 cm), and data was collected in 2020. The criteria for inclusion into the current case series included all cases where patient’s age was ≥ 14 years, with stone in the gallbladder, of size > 4 cm. Stones in the biliary tree either intra or extra hepatic were excluded. To the best of our knowledge, this is the largest case series of giant gallstones reported in the literature.

## 2 Case presentations

### 2.1 Case 1

Sudanese female, 44 years old, non-smoker presented to the general surgery clinic at our institution with one-year history of intermittent right upper quadrant colicky pain related to fatty food, not radiating, and not associated with fever or jaundice. Past medical history was significant for diabetes mellitus type 2 managed by oral hypoglycemic agents. She had no previous surgeries. There was no history of hemolytic disease. On examination, vital signs and abdominal examination were unremarkable. Investigations showed that white blood cells count and liver function tests were all within normal limits. Ultrasound of the abdomen showed normal gallbladder with no features of calculus cholecystitis, but with a single large gallstone (approximately 6 cm). The patient was diagnosed as biliary colic and was admitted for elective LC under general anesthesia. She was placed in the supine position, and a trans-umbilical camera port was inserted in addition to another 3 working ports. Intra operatively, there was some adhesions between the gallbladder and duodenum which were released by endoscopic scissors. The gallbladder wall was thick and the gallstone occupied almost all of Hartmann’s pouch which rendered grasping the gallbladder difficult. After identifying Calot’s triangle and clipping the cystic artery and duct, the gallbladder was dissected from the liver bed, put in an endobag and removed out after extension of the trans umbilical incision. The procedure was undertaken by an experienced laparoscopic general surgeon. The gallstone measured 6 × 4 × 3.3 cm. Post-operative course was uneventful and the patient was discharged home after 1 day. Post-operative histopathology showed chronic cholecystitis with intestinal metaplasia and cholesterolosis. The patient was followed up after two weeks, where she had no active complaints.

### 2.2 Case 2

Filipino female, 41 years old, non-smoker presented to the emergency department at our institution with a three-day history of right upper quadrant abdominal pain associated with multiple episodes of vomiting. The pain was not radiating and not associated with fever or jaundice. No history of chronic illnesses and no previous surgeries, and no history of hemolytic disease. On examination, she had severe right upper quadrant tenderness and positive Murphy’s sign. Liver function tests were all within normal limits, but white blood cells count was elevated (13,700 cell/L). Ultrasound of the abdomen showed large calculus within the gallbladder measuring about 4 cm with mild sludge, thick wall and pericholecystic fluid suggesting calculus cholecystitis. Patient was diagnosed as acute cholecystitis and admitted for emergency LC under general anesthesia, placed in supine position, a supra umbilical camera port was inserted in addition to another 3 working ports. Intra operatively, there was severe inflammation in which the omentum and hepatic flexure of the colon had formed a mass adherent to the gallbladder. The mass was dismantled using blunt dissection, gallbladder wall was thick and edematous, and the gallstone occupied almost all of Hartmann’s pouch. By milking the gallstone up towards the fundus, it was possible to hold Hartmann’s pouch and start the dissection. After identifying Calot’s triangle and clipping the cystic artery and duct, the gallbladder was dissected from the liver bed, put in an endobag, and removed out after minimal lateral extension of the supra umbilical incision. The procedure was undertaken by an experienced laparoscopic general surgeon. The gallstone measured 4.5 × 3.1 × 3.5 cm. Post-operative course was uneventful and the patient was discharged after two days of surgery. Post-operative histopathology showed acute cholecystitis. The patient was seen after two weeks, she had no active issues.

### 2.3 Case 3

Filipino male, 38 years old, non-smoker, presented to the general surgery clinic at our institution with intermittent right upper quadrant pain since two months radiating to the right shoulder and associated with vomiting. He had history of gallstones and a previous episode of acute cholecystitis 6 months prior that was treated conservatively. No past medical or surgical history, and no history of hemolytic disease. On examination, he had normal vital signs, and no abdominal tenderness. Ultrasound of the abdomen showed a distended gallbladder with thick wall and large calculus (about 5 cm), with few sludge balls. No evidence of pericholecystic fluid or cholecystitis. Patient was diagnosed as chronic cholecystitis and admitted for elective LC under general anesthesia. At surgery, he was placed in supine position, and a supra umbilical camera port was inserted in addition to another 3 working ports. After entrance to the peritoneal cavity, there were adhesions between the greater omentum and gallbladder. Gallbladder was distended with thick wall. As there was a dilated cystic duct that raised suspicion of common bile duct stone, an intraoperative cholangiogram was undertaken, which showed a filling defect in the distal part of the common bile duct without obstruction, and there was passage of contrast into the duodenum. Using a Dormia basket, the sludge and tiny stones were extracted from the common bile duct. A second (check) cholangiogram was then done that confirmed the smooth passage of contrast to the duodenum without any filling defect. After dissection of the gallbladder, it was put in an endobag and removed out after minimal lateral extension of the supra umbilical incision. The procedure was undertaken by an experienced laparoscopic general surgeon. The gallstone measured 4.1 × 4 × 3.6 cm. Post-operative course was smooth without any complications. Patient was discharged one day after surgery. Histopathology showed xanthomatous chronic cholecystitis. At follow up two weeks later, he had no active complaints.

**Fig. 1:**
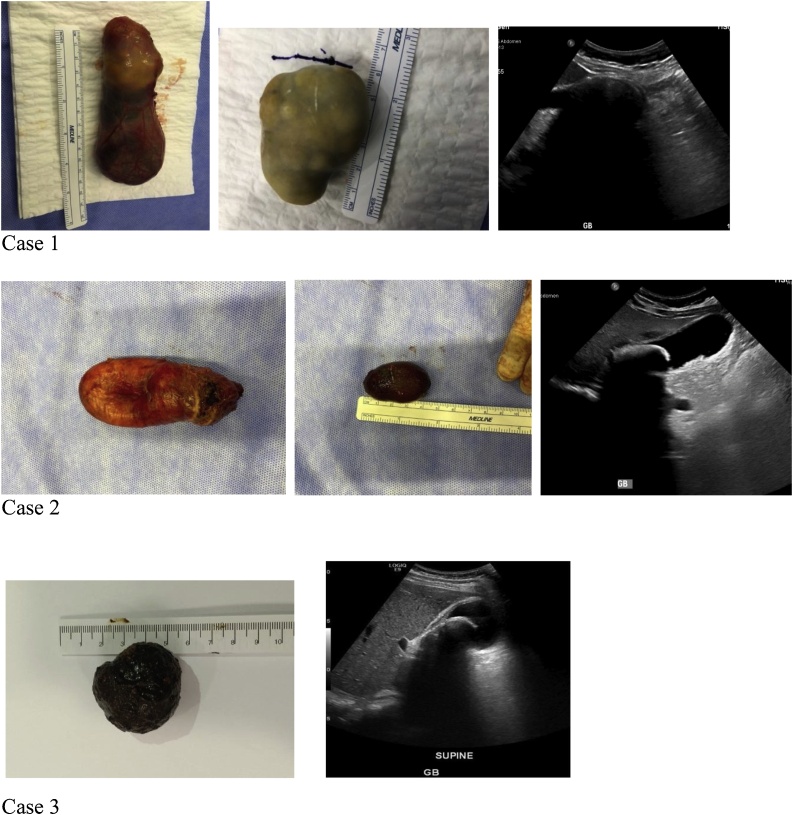
Gallbladder and Large Gallstones: three cases.

Fig. 1 shows the gallbladder and giant gallstone for each of the three cases. Table 1 provides a summary of characteristics of current case series and other reported cases of patients with large gallbladder stones.

**Table 1:** Summary of characteristics of current case series and other reported cases of large gallbladder stones identified from the review of the literature.

Case*	Sex	Age	CoMorb	AD	ST	SA	CTO	Ads	GBE	Ebag	Stone			LOS	Histopathology
											Size (cm)	Wt	Comp		
Current series Sudan	F	44	DM	BC	E	LP	N	Y	TU	Y	6 × 4 × 3.3	—	—	1	CC + IM
Current series Philippines	F	41	—	AC	ER	LP	N	Y	SU	Y	4.5 × 3.1 × 3.5	—	—	2	AC
Current series Philippines	M	38	—	CC	E	LP	N	Y	SU	Y	4.1 × 4 × 3.6	—	—	1	Xanthomatous CC
Becerra 2011 Chile	M	57	DM, HTN	AC	ER	OP	NA	—	—	—	16.8 × 7.8 × 4.1	278 g	mixed (Ch, B, Ca salts)	5	AC
Dalal 2014 India	F	38	DM	AC	E	OP	NA	—	—	—	7.4 × 5.4	72 g	mixed (Ch, B)	5	CC
Xu 2013 China	M	70	DM	AC	ER	LP	N	Y	EP	—	9.5 × 6 × 4.5	—	—	3	—
Banigo 2013 UK	F	57	—	BC	E	LP	Y	—	—	—	6 × 3 × 3.5	—	—	3	—
Igwe 2020 Nigeria	F	32	—	AC	ER	LP	N	Y	EP	—	8.2 × 7.5	—	—	7	AC
	F	62	DM, HTN	—	ER	LP	N	Y	SU	—	8 × 6	—	—	3	CC
Ekici 2007 Turkey	M	70	DM, COPD	AC	ER	LP	Y	Y	—	—	10	—	—	5	CC + DMT

* Due to space considerations only the first author is cited; — not reported; AC: Acute cholecystitis; AD: admitting diagnosis; Ads: Adhesions; BC: Biliary colic; B: bilirubin; C: Cholecystitis; Ca: calcium; CC: Chronic cholecystitis; Ch: cholesterol; CoMorb: comorbidities; Comp: composition; COPD: chronic obstructive pulmonary disease: CTO: conversion to open; DM: diabetes mellitus; DMT: diffuse metaplasia; E: Elective; Ebag: use of endobag to remove gall bladder; EP: Epigastric; ER: Emergency; F: Female; GBE: Gallbladder extraction; HTN: hypertension; IM: intestinal metaplasia; LOS: length of hospital post-op stay (day); LP: laparoscopic; M: male; N: No; NA: not applicable; OP: open; SA: Surgical approach; ST: Surgery type; SU: Supra-umbilical; TU: Trans-umbilical; Wt: Weight; y: years; Y: Yes.

## 3 Discussion

We report a case series of three patients with giant gallstones. To the best of our knowledge, this is largest case series of giant gallstones ever reported in the literature. All three cases were laparoscopically managed, and their post-operative courses were uneventful with no complications.

As for demographics, gallstones are more common in women, especially during their fertile years, probably due to increased estrogen levels which may increase cholesterol in the bile and decreased gallbladder movement, resulting in gallstone formation [1,2,11]. Our case series is in agreement, exhibiting a 2:1 female to male ratio. This ratio is despite the demographic profile in the State of Qatar, where there is 3:1 dominance of males over females due to the increase in the young male expatriate workforce in the country where immigrants comprise about 94% of Qatar’s workforce, and 70% of its population [12]. In terms of age, the frequency of gallstones increases with age, escalating after 40 years of age to become 4–10 times more likely [13]. Our patients were 38–44 years old, within the age limit of other reports (Table 1), although their ages were slightly younger than other reports, again probably due to a predominance of younger expatriate workforce in Qatar (highlighted above). As regards to ethnicity, gallstones are prevalent in developed nations, but less in the developing populations that still consume traditional diets [14]. North Americans have the highest cholelithiasis rates, South Americans also have high prevalence, intermediate prevalence rates occur in Asians and Black Americans, and sub-Saharan Black Africans have the lowest frequencies [2]. Nevertheless, our review (Table 1) shows that besides Chile, other countries where large gallstones are reported seem to be those with generally lower rates of cholelithiasis, suggesting that individual level factors might play a role in large gallstones.

In terms of associated comorbidities, dyslipidemia and diabetes mellitus are considered important associated comorbidities and risk factors for developing gallstones [15]. In support, Table 1 shows that several cases of giant gallstones had diabetes mellitus [[4], [5], [6], [7],^9^]. However, only Case 1 of our three patients reported a history of diabetes mellitus.

As for presentation, 60%–80% of gallstones are asymptomatic [16], frequently found during routine abdominal ultrasonography. Symptomatic gallstones may present as biliary pain, cholecystitis, or biliary obstruction depending on location [17]. In agreement, our cases presented as biliary colic, acute cholecystitis and chronic cholecystitis. Regarding location, gallstones exist in the gallbladder or biliary tree [18]. Gallstones can also present as gallstone ileus by migrating through a fistula between gallbladder and duodenum or small/ large bowel especially in large gallstones causing bowel obstruction [3]. Our three cases of unusually large gallbladder stones did not exhibit migration.

In connection with imaging, ultrasonography (US) is the method most often used to detect cholelithiasis and cholecystitis (90–95% specificity and sensitivity), can detect and accurately assess stone size as small as 2 mm, show thickening of the gallbladder wall, and should be routine [1,19]. US has advantages e.g. lack of ionizing radiation, noninvasiveness, option of performing a bedside examination, relatively low cost, and ability to evaluate adjacent organs [1]. For our three patients, abdominal US showed the size of the giant gallstones, with measurements close to the actual size found after surgery. Such accurate pre-operative assessment of a giant gallstone alerts the surgeon to any potential difficulty of the procedure, and the possibility of conversion to open cholecystectomy. This allows the surgeon to be prepared and to explain the potential rates of complications to the patient [5]. We were prepared in terms of surgical instruments and settings for a possible conversion to open at any point during the surgery.

In terms of size, we agree with others that consensus is required to ascertain the measurements to be used to define a gallstone as giant or large [6]. Based on composition, gallstones are classified into cholesterol (predominant entity), bilirubin (pigment), mixed (cholesterol and bilirubin), or pigment stones [20]. Our review (Table 1) shows that the composition of giant gallstones was reported in only 2 cases and both were of the mixed type (cholesterol and bilirubin) [4,7].

In terms of treatment, most gallstone patients remain asymptomatic and can be managed with watchful waiting [[21], [22], [23]]. Asymptomatic gallstones > 3 cm are at higher risk to develop gallbladder cancer and hence preventive LC is warranted [24,25]. Others reported a gallstone ileus that occurred with a large asymptomatic gallstone, and recommended prophylactic cholecystectomy for large gallstones even if asymptomatic [3]. In addition, fatal abdominal hemorrhage associated with non-traumatic perforation of gallbladder due to large gallstone is another reason for prophylactic cholecystectomy [26].

For symptomatic gallstones, LC has become the management of choice [[27], [28], [29]]. For giant gallstones, some authors believe open cholecystectomy is the choice, given the technical difficulties related to the stone's large size that could be confronted during the laparoscopic approach [4]. However, in line with others, we believe that even with giant gallstones, LC performed by an experienced laparoscopic surgeon is still the best initial approach, unless technical difficulties and inability to expose the anatomy warrants conversion to open cholecystectomy [6]. A Cochrane review (38 randomized controlled trials) suggested less complications and shorter hospital stay and convalescence in laparoscopic vs open cholecystectomy [30]. We used the laparoscopic approach for our patients without need for conversion, there were no intra- or post-operative complications, and recovery was uneventful. We support others in that laparoscopic approach is still the best initial approach for giant gallstones and should be tried [4,5], despite that some cases may end up by conversion to open cholecystectomy [8,9]. In addition to the large size of stone, other risks of conversion to open cholecystectomy is related to the surgeon, equipment factors and patient factors such as male gender, previous abdominal surgery, acute cholecystitis, thickened gallbladder wall on preoperative ultrasonography, and suspicion of common bile duct stones [31].

As for the surgical technique, giant gallstones could result in severe inflammation, adhesions and thickening of the gallbladder wall, where adhesions is an important reason for conversion of laparoscopic to open cholecystectomy [31]. In addition, giant gallstones make it difficult to grasp the gallbladder with laparoscopic instruments and expose the anatomy of Calot’s triangle [32]. We faced the same difficulties in our three cases, where the main challenge was to release the adhesions between the gallbladder and surrounding structures, and to hold the thickened and inflamed gallbladder wall by the laparoscopic grasper prior to starting dissection. Our trials of gentle milking to dislodge the giant gallstone upwards to the fundus helped to create enough space at Hartmann’s pouch to grasp the wall and start the dissection to expose Calot’s triangle. No details about such milking maneuver has been reported in previous publications of giant gallstones [4,5,7], and sometimes milking could be difficult if the stone is impacted in Hartmann’s pouch due to its large size. Others undertook a fundal incision to remove the stone and facilitate holding the gallbladder wall [4]. However, intraoperative opening of the gallbladder may increase the risk of lost stones and collections of bile in the abdomen with increased risk of abscess formation; and bile spillage in cases of incidental gallbladder cancer reduces the chances of curative resection, increases the risk of peritoneal carcinomatosis, and reduces recurrence free survival [33].

Another consideration is the site and manner of retrieval of the gallbladder out of the abdomen after cholecystectomy. As regards to site, we removed the gallbladder through the 10 mm camera port after minimal extension of the incisions. In Case 1, the camera port was trans-umbilical, while in the other two cases it was supra-umbilical. Even after extension of the incision, we believe that the supra-umbilical/ trans-umbilical retrieval approach results in better cosmetic appearance in terms of wound scar, supporting previous reports [6]. To the best of our knowledge, in LC for large gallstones without conversion to open, most retrievals were done through the epigastric port [5,6]. Only one published case reported retrieval through the umbilical port [6]. A recent systematic review of umbilical vs epigastric port retrieval showed that umbilical port retrieval may be associated with less postoperative pain in patients undergoing LC compared with epigastric port retrieval, and might also be associated with shorter gallbladder retrieval time [34]. We retrieved the gallbladder through the umbilical approach in all cases, after extension of the wound. There was no delay in retrieval time, patients had mild tolerated post-operative pain, and no wound infection. However, follow up is needed for giant gallstone patients who underwent LC in order to assess long term post-operative complications e.g. incisional hernia at site of retrieval. In terms of the manner of retrieval, for all our cases, the gallbladder was put in an endobag before taking it out of the abdomen to prevent spillage of bile or wound infection, in line with a recent meta-analysis that found that the wound infection rate was less in patients who underwent retrieval of gallbladder using a bag vs without (4.2% vs 5.9%) [35].

As regards to long term complications of large gallstones, gallstones > 3 cm have a 10.1 relative risk for the development of gallbladder cancer, compared to patients without gallstones [24,25]. Hence such patients are considered high risk and could benefit from preventive cholecystectomy [24,25]. In addition, gallstones > 3 cm carry a risk of developing biliary enteric fistula and gallstone ileus which may require surgical intervention for bowel obstruction, although the actual incidence of this progression is not known [3]. Fortunately, for the three cases we report, there was no fistula found between gallbladder and duodenum, small bowel or large bowel.

In terms of post-operative histopathology, we found chronic cholecystitis in two patients, and acute cholecystitis in one. Table 1 shows that out of the 7 cases in the review, chronic cholecystitis was reported in 3 cases [4,6,9], acute cholecystitis in 2 cases [6,7] and no histopathology was reported in 2 cases [5,8]. In addition, metaplasia was found in our Case 1, but no features of dysplasia or malignancy in all three cases. Metaplasia has been reported [9], in line with observations of metaplastic changes in the tissues of chronic cholecystitis as well as gallbladder cancer, suggesting metaplasia might be closely involved in genesis of gallbladder cancer [36].

This case series has limitations. Information on the composition of the each of the three stones would have been beneficial for the better understanding of the pathophysiology. Further research could explore the composition of giant stones in an attempt to better understand the underlying etiology of giant gallstones. Despite this, the current case series has strengths, as to the best of our knowledge, this is the largest case series of giant gallstones reported, and that it is accompanied with a thorough literature review outlining similar cases elsewhere.

## 4 Conclusions

Giant gallstones are associated with high risk of complications and LC is warranted in symptomatic and asymptomatic patients. Even for giant gallstones, LC appears to be the primary procedure of choice over open cholecystectomy and should be performed by an expert laparoscopic surgeon, taking into consideration the possibility of conversion to open in case of inability to expose the anatomy and any intraoperative technical difficulties.

## Declaration of Competing Interest

Nothing to declare.

## Funding

Nothing to declare.

## Ethical approval

Approved by Medical Research Center, Hamad Medical Corporation reference number (MRC-04-20-323).

## Consent

Verbal informed consent (in presence of a third witness) was obtained from the patients for publication of this case series and accompanying images. This is available for the Editor-in-Chief of this journal on request.

## Author contribution

Mohammad Al Zoubi: data collection, data interpretation, writing the paper. Walid El Ansari: study concept, data interpretation, writing the paper. Ahmed Al-Moudaris: data interpretation, reviewing the paper. Abdelrahman Abdelaal: study concept, data interpretation, editing the paper. All authors read and approved the final version.

## Registration of research studies

Not first in Man.

## Guarantor

Walid El Ansari: welansari9@gmail.com.

## Provenance and peer review

Not commissioned, externally peer-reviewed.
